# Economic analysis of early intervention for autistic children: findings from four case studies in England, Ireland, Italy, and Spain

**DOI:** 10.1192/j.eurpsy.2023.2449

**Published:** 2023-09-21

**Authors:** Michela Tinelli, Aine Roddy, Martin Knapp, Celso Arango, Maria Andreina Mendez, James Cusack, Declan Murphy, Roberto Canitano, Bethany Oakley, Vinciane Quoidbach

**Affiliations:** 1 Care Policy and Evaluation Centre, London School of Economics and Political Science, London, UK; 2 Atlantic Technological University, Sligo, Ireland; 3 Department of Child and Adolescent Psychiatry, Institute of Psychiatry and Mental Health, Hospital General Universitario Gregorio Marañón, IiSGM, School of Medicine, Universidad Complutense, CIBERSAM, Madrid, Spain; 4 Autistica, London, UK; 5 Kings College London, London, UK; 6 University Hospital of Siena – Azienda ospedaliero-universitaria Senese, Siena, Italy; 7 European Brain Council, Brussels, Belgium

**Keywords:** autism, children, cost-effectiveness, early intervention, family impacts, public policy

## Abstract

**Background:**

Many autistic children experience difficulties in their communication and language skills development, with consequences for social development into adulthood, often resulting in challenges over the life-course and high economic impacts for individuals, families, and society. The Preschool Autism Communication Trial (PACT) intervention is effective in terms of improved social communication and some secondary outcomes. A previously published within-trial economic analysis found that results at 13 months did not support its cost-effectiveness. We modeled cost-effectiveness over 6 years and across four European countries.

**Methods:**

Using simulation modeling, we built on economic analyses in the original trial, exploring longer-term cost-effectiveness at 6 years (in England). We adapted our model to undertake an economic analysis of PACT in Ireland, Italy, and Spain. Data on resource use were taken from the original trial and a more recent Irish observational study.

**Results:**

PACT is cost-saving over time from a societal perspective, even though we confirmed that, at 13 months post-delivery, PACT is more expensive than usual treatment (across all countries) when given to preschool autistic children. After 6 years, we found that PACT has lower costs than usual treatment in terms of unpaid care provided by parents (in all countries). Also, if we consider only out-of-pocket expenses from an Irish study, PACT costs less than usual treatment.

**Discussion:**

PACT may be recommended as a cost-saving early intervention for families with an autistic child.

## Introduction

Providing effective early support for young autistic children and their families is considered a priority across many countries [1]. Failing to address the needs of autistic individuals can have significant lifelong impacts and long-term costs for the individuals themselves, their families, health systems, and the wider society. A decade ago, per-person costs over a lifetime were estimated as £1.5 million (1.8 million euros; 2011 prices levels) in the United Kingdom for an autistic individual with learning disabilities, and £0.92 million (1.09 million euros) for an autistic individual without learning disabilities [2]. These figures prompt the question as to whether better early intervention could both reduce these costs and improve quality of life.

For children diagnosed at an early age as autistic, immediate and effective support for their social communication and development can be highly beneficial [3]. Some evidence indicates short-term benefits from various interventions, including parent–child engagement, symbolic play, and social imitation, leading to changes in individuals’ availability for learning and increased parent understanding [4]. However, developing early therapies that can effectively alter *long-term* outcomes and improve family well-being and societal outcomes has proven challenging. Additionally, there is limited evidence on positive improvements in cognitive ability, adaptive behavior, quality of life, or other important outcomes prioritized by the autism community. There is even less evidence on cost-effectiveness [5], yet such information is crucial for informing decisions on how to allocate limited healthcare and other resources.

Evidence from studies investigating the cost-effectiveness of behavior management strategies for young autistic children has been mixed, and comparisons between them is hampered by methodological differences. It has been common, for example, for studies to focus solely on economic impacts on the health and social care sectors, ignoring broader impacts (positive and negative) on other policy sectors, families, and society [5, 6]. Most studies have been short-term; capturing health and economic impacts over the longer term would provide a sounder platform for decision-making, bearing in mind the known impacts of autism across the life-course.

The Preschool Autism Communication Trial (PACT) intervention is an evidence-based that involves therapists working with parents/carers to enhance social communication in young children diagnosed at an early age as autistic [7]. PACT was the first intervention of its kind to demonstrate improvements in long-term child communication, social interaction, and other outcomes [8].

We explored the cost-effectiveness of early intervention, specifically focusing on PACT, across four European nations: England, Ireland, Italy, and Spain. These countries are beginning to make stronger commitments to support autistic individuals, despite operating within constrained public budgets. Evidence from economic evaluation can contribute helpfully to policy discussions and related decision-making.

## Methods

### The intervention and its selection

The PACT intervention is a collaborative partnership between professionals and parents/carers, aimed at enhancing social communication in autistic children. In an initial visit, the therapist and parent/carer discuss the child’s development, their specific needs, and the family’s experiences. There are 12 sessions, spaced 2 weeks apart, spanning a period of 6 months. In each session, the therapist records a short video of the parent/carer interacting with the child. The parent/carer then watches the video with the child and discusses what approaches are likely to work best for the child’s social communication. PACT was selected as the intervention of choice for the present study due to its robust evidence of effectiveness when added to treatment as usual (TAU), both short- and longer-term [7–11]. There was also a within-trial cost-effectiveness analysis at 13-month follow-up [12] but not over the 6-year period for which effectiveness evidence has been reported. For more details regarding the PACT intervention, see Appendix 1 of the Supplementary Material.

### Comparator

The comparator in the original PACT study and in this new modeling was TAU, covering a wide range of hospital and community services, including relatively high levels of contact with speech and language therapists and pediatricians [7].

### Trial participants

Participants in the original PACT study were families with a child aged 2 years to 4 years and 11 months, and meeting criteria for core autism according to internationally recognized test criteria [7]. The intervention group (PACT + TAU) comprised 74 children, whereas the control group (TAU alone) included 69 children.

### Model design

We conducted a cost-consequence analysis, which presents costs and a range of outcomes for the intervention (PACT + TAU) and comparator (TAU), rather than just a single outcome (as in cost-effectiveness analysis). This approach has been recommended when evaluating complex interventions with an array of health and non-health effects [13, 14]. Given that PACT has diverse outcomes that cannot easily be converted into monetary values or combined into a single health or other measure, the cost-consequence approach proved valuable. We took outcomes from the previously published 13-month and 6-year effectiveness studies [7, 8] and 13-month cost-effectiveness study [12] and modeled cost impacts over both periods in four European countries as part of the European Brain Council *Value of Treatment Project.* In both time periods, we considered multiple outcomes associated with the intervention’s effectiveness and its impact on the well-being and development of the children involved.

### Outcomes

In the previously reported trials, the primary outcome was severity of autism symptoms, assessed by the total score of social communication algorithm items from the Autism Diagnostic Observation Schedule-Generic (ADOS-G). A higher score on ADOS-G indicates greater severity of symptoms. The assessment was conducted by Green and colleagues at 13 months and 6 years, using updated coding [7, 8]. Notably, the results at 6 years showed significant long-term reduction in autism symptoms, and this reduction was larger compared to what was previously reported when only the social communication algorithm score was considered in the 13-month analyses.

In addition to the primary outcome, the trials also reported several secondary outcomes.

At 13 months [7]: child language; parent–child dyadic communication; autism symptoms; restricted and repetitive behaviors; social difficulties; and comorbid psychopathology.

Children who received the PACT + TAU showed greater improvement in social communication and repetitive restricted behavior symptoms compared to those who received TAU alone [7]. However, researcher-rated language skills did not show significant improvement. On the other hand, parents reported fewer difficulties in all core symptoms associated with autism (social interaction, social communication, repetitive behaviors, and restricted interests) in the PACT + TAU group compared to TAU, as well as improved everyday language [7].

At 6 years: parent–child interaction and child language and social communication rated by teachers [8]; predictors of mental health difficulties and well-being in caregivers [9]; family life experience [10]; and parental perceptions of their participation in the trial [11].

There was a statistically significant difference between the PACT + TAU and TAU groups in parent–child communication at the 6-year follow-up, indicating positive effects of the intervention. However, there were no significant between-group differences in the language composite at follow-up. Preschool assignment to PACT + TAU did not appear to be associated with rates of parental mental health difficulties or levels of mental well-being when the children were in middle childhood [9]. PACT had lasting effects on positive family life experience [10], and, overall, parents reported positive changes in their interaction and relationship with their children, as well as improvements in their children’s communication and interaction [11].

### Resource use and costs

In our economic evaluation, we considered two perspectives: a service perspective relevant to public sector policy-makers, encompassing costs of all hospital-, community-, and school-based health, social, and education services; and a societal perspective to capture broader economic implications, which included schooling and childcare costs, productivity losses (due to parents taking time off work to care for an autistic child), and informal (unpaid) care. This methodology aligns with the original PACT cost-effectiveness evaluation [12]. As part of our sensitivity analysis, we also incorporated family out-of-pocket expenses, such as aids and adaptations to the home and training courses.

To obtain data on resources used in England, we extracted aggregate data from the published PACT economic evaluation [12]. That study utilized non-parametric bootstrapping [15] to estimate summary statistics for resource use and costs. To simplify the analysis (given the complexities of finding unit costs for four different countries), we focused on resource use measures with a mean occurrence greater than 0.5 events during the 13-month follow-up period. These resource use data were then costed by applying unit costs at 2020 price levels (in Euros).

Average intervention costs per child were sourced from the PACT economic evaluation and adjusted to 2020 prices (in Euros). Other unit costs were obtained from published sources, including the PECUNIA study [16]. To calculate productivity losses, we took a human capital approach, multiplying the time parents took off work due to their child’s condition by country-specific national average salary. Informal care costs were calculated using the market price approach, which applies the amount that would be paid if the care were provided by a formal (i.e., paid) caregiver.

For our 13-month follow-up analysis, we inflated reported costs for England to 2020 prices. We then adapted the modeling to include the three additional countries: Italy, Ireland, and Spain. Cost estimates were calculated by multiplying use of resources by country-specific unit costs.

Since long-term resource use data were not available, we applied temporal extrapolation methods [17] to project short-term economic evidence from the 13-month trial over the 6-year follow-up period. It was assumed that PACT intervention costs were limited to the first 13 months to reflect typical PACT delivery practice. For education and childcare, we assumed that nursery school costs were applicable for the initial 2 years of the model only. From the third year onward, we assumed that all children were in primary school education, and the time previously allocated to nursery was costed as school time instead. Fixed estimates of yearly costs, as per the PACT trial, were applied for health-, social care-, education-, and family-related cost categories, and these assumptions were applied across all four countries.

Values of costs and benefits were adjusted for the time they occurred using discounting, applying a rate of 3.5%. This allowed for accounting for the time preference of costs and benefits over the 6-year follow-up period.

### Sensitivity analysis

We performed several deterministic sensitivity analyses.

*Sensitivity analysis 1: Health and social care, education, and family impacts.* We looked at individual categories of costs, varying their relative unit costs estimates in the model by a given amount (±20%, ±30%, and ±50%) and examining impacts on results.

*Sensitivity analysis 2: Family impacts (parental productivity loss and informal care).* We varied productivity loss hours and informal care hours according to different stages of the model. For years 1 and 2 (child aged 4 or 5 years), we considered the same productivity loss hours and informal care hours as per the PACT trial, whereas for years 3–6 (child aged 6 years or older), we assumed that yearly productivity loss hours and informal care hours would decrease by 20%, based on findings from a Scottish study [18].

*Sensitivity analysis 3: Family impacts (parental out-of-pocket expenses) for the Irish case study.* From the PACT trial, we know that the difference in out-of-pocket expenses was not statistically significant when considering aids and adaptations to the home, training courses, and so forth [12]. For the Irish case study, new data on TAU were sourced from a mixed-methods observational study on the economic impact and unmet needs of Irish autistic children [19, 20]. The study considered a cohort aged 2–5 years, comparable in age to the PACT sample at baseline. Cost figures were inflated to 2020 prices and included: living costs, care and assistance, education, healthcare, travel, training/support, and autism assistance dog. We compared parental out-of-pocket costs for the PACT + TAU group sourced from the PACT trial with matching items of cost data extracted from the Irish study.

## Results

### Costs

**Figure 1.**
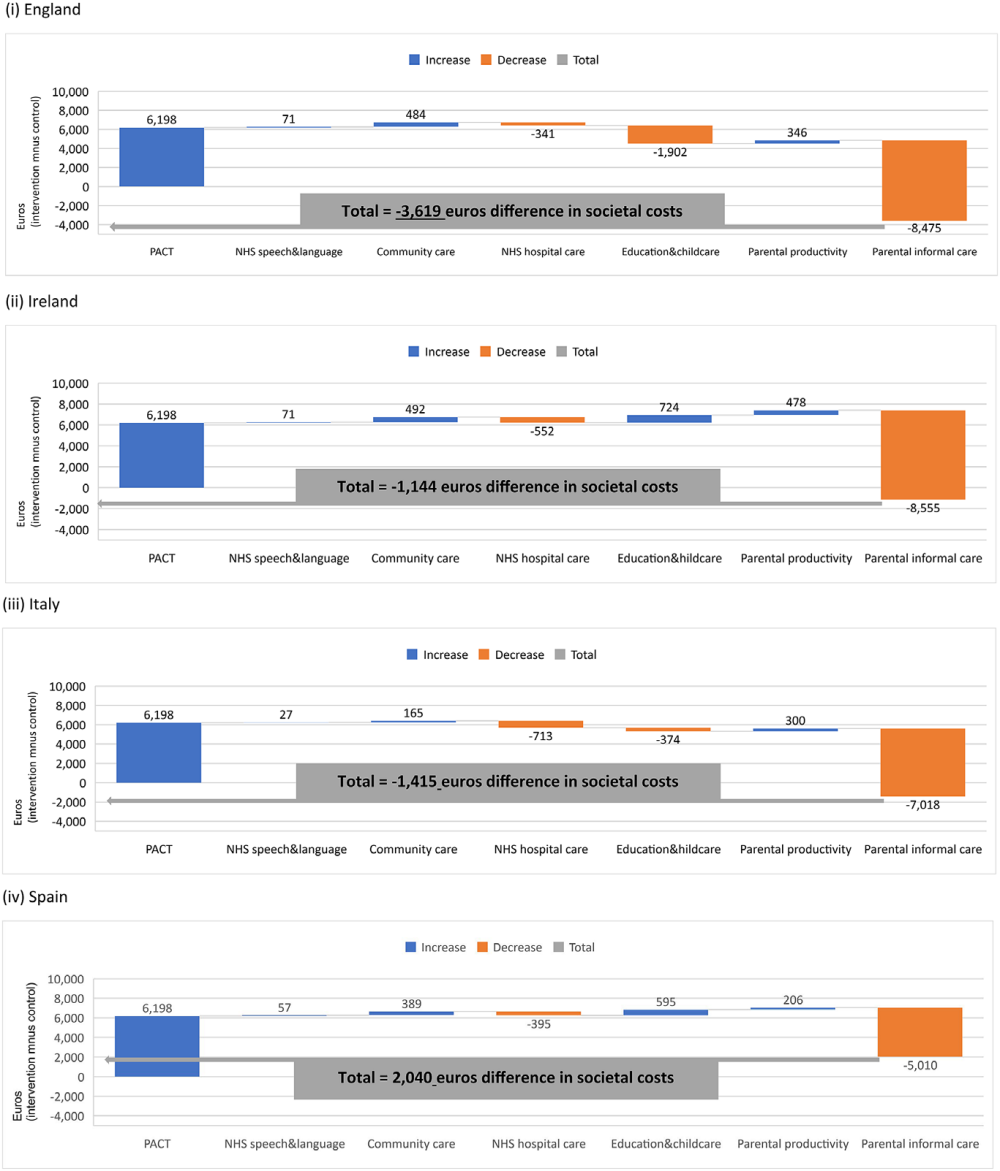
Understanding the cumulative effect of introducing individual items of costs to build a final aggregate value of the difference in total societal costs (intervention minus control) at 13 months. Cost items reported on the *y*-axis: Preschool Autism Communication Trial (PACT), healthcare speech and language therapy, community health and social services, hospital-based health services, education and childcare, parental productivity, and parental informal care. This waterfall chart helps in understanding how an initial value for the difference in delivery costs between groups (PACT + TAU vs. TAU) is increased (cost items) and decreased (cost items in orange) by adding a series of cost items (health-, social care-, education-, and family-related cost categories), leading to a final aggregate value of the difference in total societal costs at 13 months (total value indicated by the gray line).

Cost findings presented here are derived from our model. Figure 1 shows how an initial value for the difference in delivery costs between groups is increased and decreased by adding a series of cost items (for various health-, social care-, education-, and family-related cost categories), leading to a final aggregate difference in societal costs at 13 months. The initial cost difference is shown in blue (indicating a positive value of 6,198 euros for England). As reported by the original trial economic evaluation, service costs for England at 13 months were significantly higher for PACT + TAU than TAU alone. If we consider the healthcare service perspective, the difference in total costs between groups was 5,928 euros (7,651 euros PACT + TAU vs. 1,723 euros TAU). This difference decreased to 4,510 euros, when we consider a broader service perspective (including healthcare, education, and social care).

Overall, the difference in total societal costs between groups was smaller (−3,619 euros; 95,689 euros PACT + TAU vs. 99,308 euros TAU) due to lower informal care rates for PACT + TAU. The difference in total societal costs is indicated by the gray line below zero. The original trial evaluation reported that this difference was non-statistically significant [18]. Similar results applied to other nations. The breakdown per individual cost item (for the provider and family perspectives per group) across nations is presented in Table 1.

**Table 1.** Mean costs (Euros, 2020) at 13 months

	PACT + TAU	TAU
ENGLAND	Mean costs (Euros)	Mean costs (Euros)
PACT	6,198	0
Healthcare speech and language therapy	689	618
Other community health, education, and social services	1,652	1,168
Hospital-based health services	764	1,105
Education and childcare	6,937	8,839
Parental productivity losses	796	450
Parental informal care	78,653	87,128
Total service perspective (health, education, and social services)	**16,240**	**11,730**
Total societal perspective (including parental productivity losses and informal care)	**95,689**	**99,308**
IRELAND		
PACT	6,198	0
Healthcare speech and language therapy	686	615
Other community health, education, and social services	1,544	1,052
Hospital-based health services	438	990
Education and childcare	10,408	9,684
Parental productivity losses	1,099	621
Parental informal care	79,398	87,953
Total service perspective (health, education, and social services)	**19,274**	**12,341**
Total societal perspective (including parental productivity losses and informal care)	**99,771**	**100,915**
ITALY		
PACT	6,198	0
Healthcare speech and language therapy	256	229
Other community health, education, and social services	520	355
Hospital-based health services	296	1,009
Education and childcare	5,040	5,414
Parental productivity losses	690	390
Parental informal care	65,134	72,152
Total service perspective (health, education, and social services)	**12,310**	**7,007**
Total societal perspective (including parental productivity losses and informal care)	**78,134**	**79,549**
SPAIN		
PACT	6,198	0
Healthcare speech and language therapy	2,642	2,529
Other community health, education, and social services	6,052	4,143
Hospital-based health services	2,405	4,416
Education and childcare	112,042	114,408
Parental productivity losses	2,642	2,529
Parental informal care	6,052	4,143
Total service perspective (health, education, and social services)	**25,864**	**19,020**
Total societal perspective (including parental productivity losses and informal care)	**72,843**	**70,803**

Abbreviation: PACT, Preschool Autism Communication Trial; TAU, treatment as usual.

**Figure 2.**
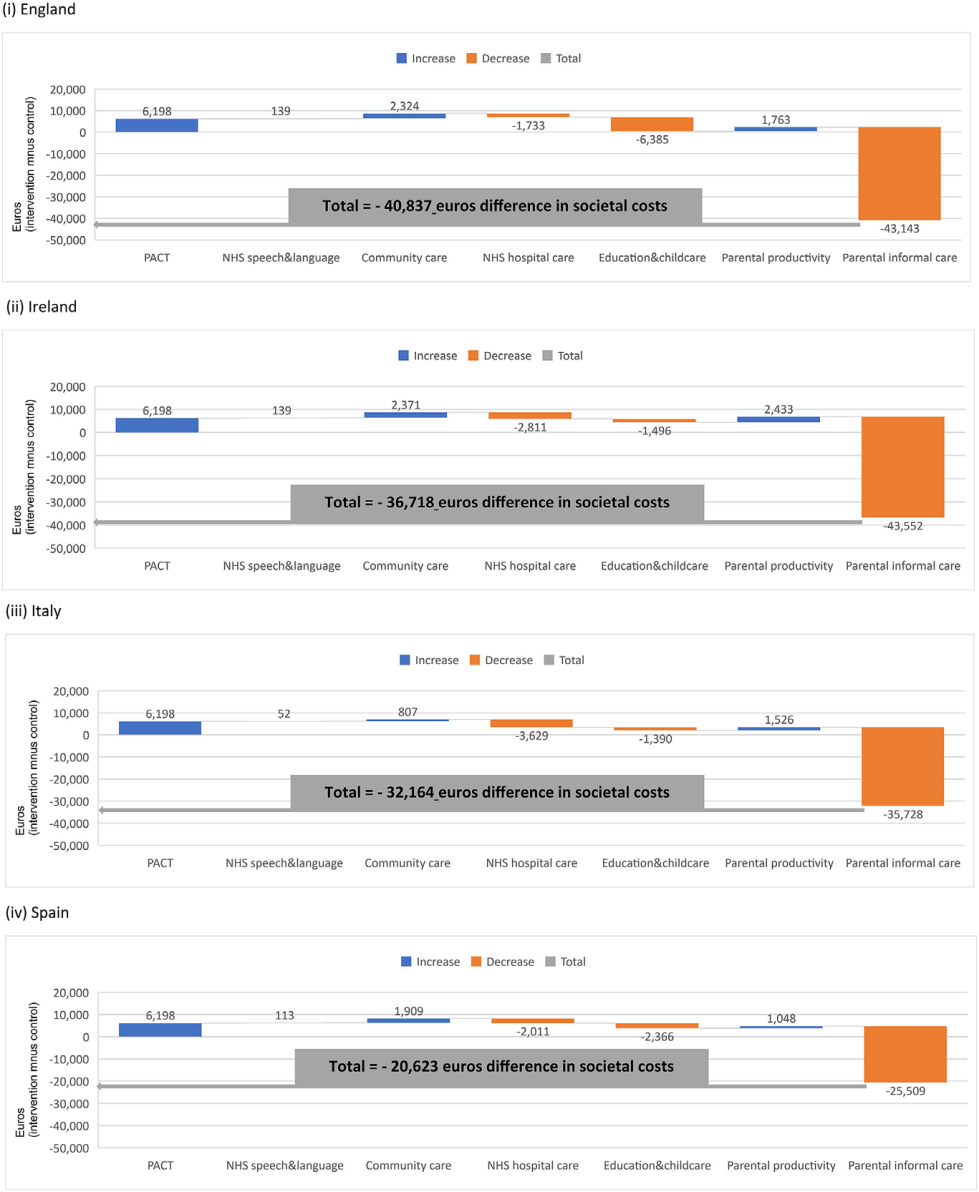
Understanding the cumulative effect of introducing individual items of costs to build a final value for the difference in total societal costs (intervention minus control) at 6 years. Cost items reported on the *x-*axis: Preschool Autism Communication Trial (PACT), healthcare speech and language therapy, community health and social services, hospital-based health services, education and childcare, parental productivity, and parental informal care. This waterfall chart helps in understanding how an initial value for the difference in delivery costs between groups (PACT intervention vs. control) is increased (cost items in blue) and decreased (cost items in orange) by adding a series of cost items (for various health-, social care-, education-, and family-related cost categories), leading to a final aggregate value of the difference in total societal costs at 6 years (total value indicated by the gray line).

At 6 years, for England, service costs remained significantly higher for PACT + TAU than TAU alone (positive value of 6,198 in blue; Figure 2). After adding the cost estimates for health, total costs for PACT + TAU were still higher than TAU alone (positive value of 4,604 euros). When we consider a broader service perspective, the difference in costs between groups was 543 euros (Table 2). Due to lower informal care rates for PACT + TAU compared with TAU (corresponding to a saving of −43,143 euros), the difference in societal costs translated into a saving of −40,837 euros (Figure 2). Similar results applied to other nations.

**Table 2.** Mean costs (Euros, 2020) at 6 years

	PACT + TAU	TAU
ENGLAND	Mean costs (Euros)	Mean costs (Euros)
PACT	6,198	0
Healthcare speech and language therapy	3,285	3,146
Other community health, education, and social services	8,270	5,946
Hospital-based health services	3,890	5,623
Education and childcare	39,461	45,846
Parental productivity losses	4,054	2,291
Parental informal care	400,409	443,552
Total service perspective (health, education, and social services)	**61,104**	**60,561**
Total societal perspective (including parental productivity losses and informal care)	**465,567**	**506,403**
IRELAND		
PACT	6,198	0
Healthcare speech and language therapy	3,272	3,133
Other community health, education, and social services	7,728	5,357
Hospital-based health services	2,231	5,042
Education and childcare	61,409	62,905
Parental productivity losses	5,594	3,161
Parental informal care	404,202	447,754
Total service perspective (health, education, and social services)	**80,838**	**76,437**
Total societal perspective (including parental productivity losses and informal care)	**490,634**	**527,353**
ITALY		
PACT	6,198	0
Healthcare speech and language therapy	1,219	1,167
Other community health, education, and social services	2,616	1,809
Hospital-based health services	1,509	5,138
Education and childcare	19,662	21,052
Parental productivity losses	3,510	1,984
Parental informal care	331,585	367,313
Total service perspective (health, education, and social services)	**31,204**	**29,166**
Total societal perspective (including parental productivity losses and informal care)	**366,299**	**398,463**
SPAIN		
PACT	6,198	0
Healthcare speech and language therapy	2,642	2,529
Other community health, education, and social services	6,052	4,143
Hospital-based health services	2,405	4,416
Education and childcare	112,042	114,408
Parental productivity losses	2,409	1,361
Parental informal care	236,752	262,261
Total service perspective (health, education, and social services)	**129,339**	**125,496**
Total societal perspective (including parental productivity losses and informal care)	**368,496**	**389,119**

Abbreviation: PACT, Preschool Autism Communication Trial; TAU, treatment as usual.

#### Sensitivity analysis

*Sensitivity analysis 1: Health and* *social care, education, and family impacts (parental productivity loss and informal care).* When we varied unit cost estimates in the 6-year model by a given amount, the difference between groups was comparable, regardless of the type of costs considered or the magnitude of the change applied (Appendix 3 of the Supplementary Material).

*Sensitivity analysis 2: Family impacts (parental productivity loss and informal care).* Although we varied productivity loss hours and informal care hours according to different stages of the model, total societal costs between groups remained similar across nations (Appendix 4 of the Supplementary Material).

*Sensitivity analysis 3: Family impacts (parental out-of-pocket expenses).* When we compared mean parental out-of-pocket estimates from the PACT intervention [12] with the corresponding estimates from Roddy and O’Neill [19], the difference between groups was −7,903 euros (1,696 vs. 9,599 euros), with contrast in parental out-of-pocket expenses of more than 80% (Table 3).

**Table 3.** Out-of-pocket expenditure (Euros, 2020) reported per child aged 2–5 years in Ireland

Cost item	Mean costs (Euros)	SD
Living costs	**3,652**	**4,113**
Special diet	990	1,112
Special clothing	190	407
Continence care, e.g., nappies	352	417
Replacing/repairing damage	292	568
Home adaptations	775	2,434
Extra heat	219	370
Extra electricity	355	619
Laundry	283	503
Telephone	197	313
Care and assistance costs	**1,374**	**2,231**
Childcare/carer during the school term	1,106	1,768
Childcare/carer during the holidays	269	583
Respite care	0	0
Special activities’ costs		
Autism-friendly activities	556	711
Educational costs	**89**	**881**
Specialized education	273	661
Therapeutic toys and sensory equipment	367	384
Electronic items, e.g., iPad	256	249
Medical costs	**1,915**	**2,543**
Out-of-pocket expenses for GP visit	176	448
Out-of-pocket for specialists	535	1,395
Out-of-pocket expenses for medication/supplements	174	238
Out-of-pocket expenses for private therapeutic interventions and assessment	968	1,593
Out-of-pocket expenses for hospital patient fee	62	198
Travel costs	**762**	**1,007**
Fuel/transport/parking costs	746	989
Accommodation	16	64
Training/support costs	**221**	**471**
Skills training course(s)/workshops	129	316
Counseling	91	377
Autism assistance dog’s costs		
Training/veterinary bills/feeding	77	390
Other costs	**146**	**719**
TOTAL OUT-POCKET EXPENDITURE	**9,599**	**8,039**

*Note*: Non-parametric bootstrapping method was used to estimate summary statistics.

## Discussion

Despite not being considered cost-effective at the 13-month post-delivery mark, the PACT intervention is likely to become cost-saving over time, particularly when taking a societal perspective into account. Our cost-consequence analysis confirmed that, at 13 months, the improved effectiveness of PACT (enhanced social communication and reduced repetitive restricted behavior symptoms, along with fewer difficulties in core autism symptoms) did not result in noticeable changes in costs for public services or society as a whole across the nations studied.

However, at the 6-year follow-up, the long-term reduction in autism symptoms, improved parent–child communication, positive family life experiences, and enhanced parent–child interactions were accompanied by a reduction in downstream costs, particularly those related to parental informal care. This reduction in costs canceled out the initial intervention costs associated with PACT and revealed promising cost-saving trends when taking a societal perspective, applicable to all countries studied.

Furthermore, our analysis showed that PACT can lead to cost-saving outcomes when considering out-of-pocket expenditures incurred by families in Ireland. Family out-of-pocket expenses in Ireland were notably higher compared to those reported by families participating in the randomized controlled trial in England. Unfortunately, we did not have equivalent data for the other countries to perform similar comparisons.

### Strengths and weaknesses

In our economic evaluation, the effectiveness evidence was obtained from a previous randomized controlled trial and a follow-up study. However, data on the use of resources were limited to the first 13 months. To project economic estimates over a longer time frame, we utilized extrapolation techniques. While the results suggest promising cost-saving trends at the 6-year follow-up, access to longer-term trial data would be necessary to further validate and confirm our findings.

Resource use data and PACT delivery costs were extracted from the original PACT trial conducted in England. Unit cost data were primarily sourced from local tariffs and the PECUNIA toolkit [16]. However, to fill gaps and validate assumptions, we sought inputs from national experts from the three countries included in the study (Italy, Ireland, and Spain), chosen for their knowledge and experience with local healthcare systems and service delivery.

For the sensitivity analysis regarding parental out-of-pocket expenses, we had more up-to-date Irish data for the TAU group from a population study [19]. In contrast, parental out-of-pocket estimates for the PACT group were sourced from the original PACT trial [12]. Although data were extracted from separate studies examining service provision under different conditions, both studies considered cohorts of children with similar characteristics and collected similar categories of out-of-pocket expenses.

### Comparison with other studies

Few economic evaluations have looked at interventions for autistic individuals. Previous reviews, such as those conducted by Romeo et al. [21] and Sampaio et al. [5], have highlighted the scarcity of economic evidence in this area, particularly cost-effectiveness analyses and other evaluations. Among the studies that we reviewed when looking for early interventions for autistic children and their families, the economic evaluation within the original PACT trial stood out as one of the few that investigated cost-effectiveness using robust methods [12]. PACT intervention was found to be effective in improving social communication and other outcomes beyond the intervention period, but the associated economic evaluation concluded that it was not cost-effective at the 13-month follow-up.

Another study reviewed by Sampaio et al. [5], conducted by Penner et al. [22], reported that early intervention for children thought to be autistic might be associated with cost-savings compared to current practice in Canada. This finding also suggests that targeted early interventions for autistic children can have long-term cost-saving implications.

The modeling in the study reported in this paper expands on the evidence from the original PACT trial by projecting the economic implications over the longer period of 6 years and also across four European countries (England, Ireland, Italy, and Spain). Over this extended time horizon, PACT may become less expensive from both public service and societal perspectives in all four countries. This suggests that the long-term benefits and potential cost-saving trends associated with PACT warrant consideration by decision-makers when allocating resources, whether to support autistic children and their families or for wider healthcare and other purposes.

### Implications for policy and practice

The results from our modeling study, particularly the sensitivity analysis using Irish out-of-pocket expenditure estimates, reveal that PACT, which has already been shown to be effective, could saves costs over the longer term. (There is no reason to believe that the cost savings up to the 6-year point would be canceled out by later cost increases for the PACT + TAU group relative to the TAU only group.) This finding has important implications for policy and practice development, especially in Ireland, where resources, staff skills, acceptability, and reach to children in need must be taken into consideration.

It is also important to note that PACT does not appear to be cost-saving from the perspective of public services, but only when economic effects on families are taking into account. If policy and practice communities fail to factor these wider societal costs and savings into their decision-making, there is a risk that effective interventions such as PACT would not get the resources they deserve.

PACT is considered low-intensity compared to other early interventions, and it appears that it should be affordable for the healthcare sector. Both staff and families can access online training, which helps improve accessibility. However, challenges such as the current backlog of clinic appointments in Ireland, as evidenced by the substantial number of children on waiting lists for speech and language assessments and therapy [23], and the long waiting lists for autism diagnosis in the United Kingdom [24], may limit timely access to services for autistic children and their families.

The pressing need for appropriate support for autistic individuals and their families is well understood by both affected families and clinical, education, and other professionals. With the right support, autistic individuals can be empowered with more life opportunities and achieve better outcomes. The 2021 Practice Guidance from the European Society for Child and Adolescent Psychiatry recognizes the importance of early access to appropriate interventions and education for autistic children [25] Among the developmentally based therapies designed to facilitate social communication between very young children and their parents, PACT is suggested as one of the most rigorously evaluated approaches.

The Lancet Commission on the Future of Care and clinical research in Autism also identifies PACT as a key parent-mediated therapy for children. It has been successfully implemented in high-resource settings and adapted to support evidence-based care in low-resource settings worldwide [4]. Our new evidence on the cost-saving potential of PACT provides strong economic support to these recommendations, further highlighting the importance of early, evidence-based interventions for autistic individuals.

## Supporting information

Tinelli et al. supplementary material Tinelli et al. supplementary material

Tinelli et al. supplementary material Tinelli et al. supplementary material

## Data Availability

The data that support the findings of this study are reported in the main text and the Supplementary Material.

## Supplementary material

The supplementary material for this article can be found at http://doi.org/10.1192/j.eurpsy.2023.2449.
