# Supplementary material for: Economic analysis of early intervention for autistic children: findings from four case studies in England, Ireland, Italy, and Spain
Source: Eur Psychiatry. 2023 Sep 21;66(1):e76. doi: 10.1192/j.eurpsy.2023.2449 (PMC10594363; doi:10.1192/j.eurpsy.2023.2449)
Supplement: Tinelli et al. supplementary material [file S0924933823024495sup001.docx]

**Supplementary material**

**Appendix 1: Selecting the Intervention for the economic model**

We searched the literature to map evidence on early intervention of autistic children. We worked with experts (including representatives from clinical and patient groups) to agree on which interventions were to be the focus of our exploration of economic arguments. We were interested in whether interventions were considered to be effective, cost-effective, affordable and acceptable to families, as well as their potential reach into the prevalent population, and whether suitable staff were likely to be available in the study countries (NICE, 2011).

The interventions considered were the Preschool Autism Communication Trial (PACT; Green et al., 2010), Early Start Denver Model (Rogers, et al., 2010), Joint Attention Symbolic Play Engagement and Regulation programme (Kasari et al., 2014; Shire et al., 2017), Focus Playtime Intervention (Siller et al., 2013), Early and Intensive Behavioural Intervention (Lovaas et al., 1987), and Applied Behavior Analysis (Dawson, 2008) derived from Lovaas.

PACT was the preferred intervention for our study. It scores highly in terms of effectiveness, affordability for the healthcare sector, availability of staff skills, acceptability by practitioners, autistic people and families, and reach to children in need (Green et al., 2010; Byford et al., 2015; PACT Training, 2021). It is an early social communication intervention based on collaborative work with autistic people, parents and carers, and the first to have demonstrated long-term improvements in autism symptoms after one-year therapy within a clinical trial. Although the economic evidence from the PACT trial showed that the intervention at 13 months is not cost-effective compared with control, it is the only intervention of this type to date to have shown sustained symptom improvement in autistic children over the longer term: children who received PACT showed greater improvement in social communication and repetitive restricted behaviour symptoms when compared to those who had usual care alone. Significant gains in parent-child interaction (shown in other analyses to mediate child symptom changes) were seen at 6 months. Researcher-rated language skills were an area that did not improve. Parents reported fewer difficulties in all the core symptoms associated with autism: social interaction, social communication, repetitive behaviours and restricted interests in the PACT group, compared to usual care, as well as improved everyday language.

These relative improvements were still evident when the children were followed up 6 years after the end of the original intervention (Pickles et al., 2016). About 80% of the original 152 trial participants were assessed when they reached age 8-10 years by researchers who did not know if a child had received the PACT therapy or not. The relative improvement in autism symptom severity, child social engagement, meaningful communication initiations, and reduced restricted repetitive and stereotyped behaviours continued for an extended period.

The original economic analysis within the PACT trial (Byford et al., 2015) looked at 152 preschool autistic children randomly assigned to treatment as usual (TAU) or PACT added to TAU (PACT+TAU). The primary outcome was the severity of autism symptoms at a 13-month follow-up. Economic data included costs associated with health, education and social services, childcare, parental productivity losses and informal care. The cost-effectiveness of PACT+TAU was explored in terms of costs per unit improvement in the proportion of children who demonstrated a clinically meaningful ADOS-G improvement (equivalent to an improvement in ADOS-G score between baseline and follow-up of ≥4 points; Stinnett, et al., 1998). PACT+TAU generated improvements in clinical outcomes, but it was not found to be cost-effective. Service costs were significantly higher for PACT+TAU, but the difference in societal costs was smaller and non-significant due to lower informal care rates for PACT+TAU.

Although the original economic modelling showed PACT as not cost-effective, it is likely to be considered as affordable for the healthcare sector: its mean cost is £4105 (4874 euros) per participant over the 13-month follow-up period (UK 2007 price levels; Byford et al., 2015) and staff skills needed for delivery are broadly available across high-income countries.

A not-for-profit community interest company, Interaction Methods for Paediatric Autism Communication Therapy (2021), was set up to deliver PACT training to professionals and support the implementation of PACT through an international programme including England, Italy and Spain among others. Children in need can be reached through a wide range of carers and professionals (who can easily be trained online). This low-intensity approach is noted in NICE (2022) guidance for therapies for autistic people in the UK and is being implemented through professional training in the UK and elsewhere. It is also accepted by the international community of practitioners, and evidence of its effectiveness and cost-effectiveness has been published in well-regarded journals (Green et al. 2010; Byford et al., 2015; Pickles et al.,2016; Salomone et al., 2017; Leadbitter et al., 2018; Leadbitter et al., 2020). Following the selection of the preferred intervention (PACT) we developed the economic model.

**References for appendix 1**

Byford S, Cary M, Barrett B, Aldred CR, Charman T, Howlin P, Hudry K, Leadbitter K, Le Couteur A, McConachie H, Pickles A, Slonims V, Temple KJ, Green J; PACT Consortium. Cost-effectiveness analysis of a communication-focused therapy for pre-school children with autism: results from a randomised controlled trial. BMC Psychiatry. 2015 Dec 21;15:316.

Dawson G. Early behavioral intervention, brain plasticity, and the prevention of autism spectrum disorder. Dev Psychopathol. 2008 Summer;20(3):775-803.

Green J, Charman T, McConachie H, Aldred C, Slonims V, Howlin P, Le Couteur A, Leadbitter K, Hudry K, Byford S, Barrett B, Temple K, Macdonald W, Pickles A; PACT Consortium. Parent-mediated communication-focused treatment in children with autism (PACT): a randomised controlled trial. Lancet. 2010 Jun 19;375(9732):2152-60.

Kasari C, Siller M, Huynh LN, Shih W, Swanson M, Hellemann GS, Sugar CA. Randomized controlled trial of parental responsiveness intervention for toddlers at high risk for autism. Infant Behav Dev. 2014 Nov;37(4):711-21.

Lovaas OI. Behavioral treatment and normal educational and intellectual functioning in young autistic children. J Consult Clin Psychol. 1987 Feb;55(1):3-9.

National Institute for Health and Care Excellence. Supporting investment in public health: Review of methods for assessing cost-effectiveness, cost impact and return on investment, 2011. Retrieved January 6, 2023, from: https://www.nice.org.uk/media/default/About/what-we-do/NICE-guidance/NICE-guidelines/Public-health-guidelines/Additional-publications/Cost-impact-proof-of-concept.pdf.

National Institute for Health and Care Excellence. Autism spectrum disorder in under 19s: support and management. Clinical guideline 2022 [CG170]. Retrieved January 6, 2023, from: <https://www.nice.org.uk/guidance/cg170>.

PACT Training (2021). Information for professionals. Retrieved January 6, 2023, from: [https://www.pacttraining.co.uk/information-for-professionals](https://www.pacttraining.co.uk/information-for-professionals/).

Pickles A, Le Couteur A, Leadbitter K, Salomone E, Cole-Fletcher R, Tobin H, Gammer I, Lowry J, Vamvakas G, Byford S, Aldred C, Slonims V, McConachie H, Howlin P, Parr JR, Charman T, Green J. Parent-mediated social communication therapy for young children with autism (PACT): long-term follow-up of a randomised controlled trial. Lancet. 2016 Nov 19;388(10059):2501-2509.

Rogers SJ, Dawson G. Early Start Denver Model for young children with autism: promoting language, learning, and engagement. 2010 New York, NY: Guilford Press.

Siller M, Hutman T, Sigman M. A parent-mediated intervention to increase responsive parental behaviors and child communication in children with ASD: a randomized clinical trial. J Autism Dev Disord. 2013 Mar;43(3):540-55.

Stinnett AA, Mullahy J. Net health benefits: a new framework for the analysis of uncertainty in cost-effectiveness analysis. Med Decis Making. 1998 Apr-Jun;18(2 Suppl):S68-80.

Interaction Methods for Paediatric Autism Communication Therapy (IMPACT). Retrieved January 6, 2023, from https://www.pacttraining.co.uk/about-pact-training/mission-statement.

**Appendix 2: Unit costs**

**Table A1: England**

|  | Cost item | Euros (2020) | Source |
| --- | --- | --- | --- |
| Speech and language therapy | PACT sessions - From the trial we know that: the average cost of a PACT session was estimated to be £264 and the total cost of the PACT intervention was £4,105 per child, on average; the mean number of sessions attended was 16 out of a maximum of 19. | 431 | 264 (2007 values) from Byford et al (2015)  £375 (2020 values) converted to euros |
|  | Healthcare speech and language therapy sessions | 48 | Curtis et al (2020) |
| Other community health, education and social services | General practitioner contacts | 44 | Curtis et al (2020) |
|  | General practice nurse contacts | 44 | Curtis et al (2020) |
|  | Health visitor contacts | 57 | Band 6 £49 per hour, see Curtis et al (2020)) |
|  | Community paediatrician contacts | 256 | Curtis et al (2020) |
|  | Clinical psychologist contacts | 137 | Curtis et al (2020) |
|  | Social worker contacts | 60 | Band 6 £49 per hour, see Curtis et al (2020) |
|  | Occupational therapist contacts | 164 | Curtis et al (2020) |
|  | Special Education Needs Coordinator contacts | 42 | £36 per hour Prospect UK (https://www.prospects.ac.uk/) |
|  | Portage worker contacts | 36 | £31 per hour National Career Services (https://nationalcareers.service.gov.uk) |
|  | Osteopath contacts | 45 | Band 5 £39 per hour  Health Careers (https://www.healthcareers.nhs.uk) |
|  | Art worker contacts | 57 | Band 6 £49 per hour  Health Careers (https://www.healthcareers.nhs.uk) |
|  | Voluntary sector service contacts | 45 | About £39 per hour  ThirdSector (https://www.thirdsector.co.uk) |
|  | Voluntary sector telephone helpline calls | 45 | About £39 per hour  ThirdSector (https://www.thirdsector.co.uk) |
|  | Other community service contacts | 45 | About £39 per hour band 5, see Curtis et al (2020) |
| Hospital-based health services | Hospital nights | 396 | National Cost Collection for the NHS (https://www.england.nhs.uk/national-cost-collection/) |
|  | Outpatient visits | 256 | Curtis et al (2020) |
|  | Accident and emergency visits | 258 | Curtis et al (2020) |
| Education and childcare | Mainstream nursery weeks |  | Free if part of the state school system. |
|  | Specialist nursery weeks | 292 | Day nursery (50 hours for a child under 2): £252 per week. Money Helper (https://www.moneyhelper.org.uk/) |
|  | Mainstream playgroup weeks | 153 | 9am to 3pm: £132 per week (https://www.bishopthorpe-playgroup.org.uk) |
|  | Specialist playgroup weeks | 327 | we applied that there is an average higher cost of £5 an hour for every child with SEND (https://www.gov.uk/government/organisations/department-for-education). We considered about 6 hours a day for a week (5 days) = 30 hours. This equates to about £150 additional costs compared to mainstream playgroups. |
|  | Mainstream school weeks | 77 | 2018 Annual Report on Education Spending in England (https://ifs.org.uk). Yearly costs per child were £3,200 in 2017–18. This equates to about £3443 per year in 2020- 39 weeks in a school year; we assumed about £66 per week. |
|  | Specialist school weeks | 151 | Maintained Specialist Schools. We assumed about 130 per week (https://www.gov.uk/government/organisations/department-for-education) |
|  | Home tutor weeks | 70 | Two sessions per week, £30 each Prospect UK (https://www.prospects.ac.uk) |
|  | Childminder week | 86 | (Money Helper https://www.moneyhelper.org.uk/) |
| Parental productivity losses and informal care | Productivity loss total hours | 18 | Mean hour earning (2019) (https://www.ons.gov.uk/). about £15 per hour (584.9/40) (2019 values) updated to £15.23 (2020 values). |
|  | Informal care hours per day | 24 | NHS Paying for your care (self-funding; https://www.nhs.uk). A typical hourly rate for a carer to come to your home is around £20 (2018 values). This is updated to£20.82 (2020 value). |

**Table A2: Ireland**

|  | Cost item | Euros (2020) | Source |
| --- | --- | --- | --- |
| Speech and language therapy | PACT sessions - From the trial we know that: the average cost of a PACT session was estimated to be £264, and the total cost of the PACT intervention was £4,105 per child, on average; the mean number of sessions attended was 16 out of a maximum of 19. | 431 | see English data |
|  | Healthcare speech and language therapy sessions | 48 | Curtis et al (2020) |
| Other community health, education, and social services | General practitioner contacts | 44 | Curtis et al (2020) |
|  | General practice nurse contacts | 44 | Curtis et al (2020) |
|  | Health visitor contacts | 26 | Irish Nurses & Midwives Organisation (https://www.inmo.ie) |
|  | Community paediatrician contacts | 256 | Curtis et al (2020) |
|  | Clinical psychologist contacts | 137 | Curtis et al (2020) |
|  | Social worker contacts | 60 | Band 6 £49 per hour; see Curtis et al (2020) |
|  | Occupational therapist contacts | 163 | Curtis et al (2020) |
|  | Special Education Needs Coordinator contacts | 42 | £36 per hour  Prospect UK (https://www.prospects.ac.uk) |
|  | Portage worker contacts | -- | Not applicable in Ireland |
|  | Osteopath contacts | 70 | The Osteopathic Council of Ireland (https://www.osteopathy.ie) |
|  | Art worker contacts | 40 | - Payscale (https://www.payscale.com) |
|  | Voluntary sector service contacts | 45 | About £39 per hour  ThirdSector (https://www.thirdsector.co.uk) |
|  | Voluntary sector telephone helpline calls | 19 | Indeed Ireland (<https://ie.indeed.com>) |
|  | Other community service contacts | 45 | About £39 per hour band 5; Curtis et al. (2020) |
| Hospital-based health services | Hospital nights | 909 | Office of the Regional Director of Operations (2020) (https://www.hse.ie) |
|  | Outpatient visits | 100 | Irish Nurses & Midwives Organisation 2020 (https://www.inmo.ie) |
|  | Accident and emergency visits | 257 | Curtis et al (2020) |
| Education and childcare | Mainstream nursery weeks | 183 | Government of Ireland (2019) (https://www.gov.ie) |
|  | Specialist nursery weeks |  | Not applicable in Ireland |
|  | Mainstream playgroup weeks | 73 | Government of Ireland (2019) (https://www.gov.ie) |
|  | Specialist playgroup weeks | -- | Not applicable in Ireland |
|  | Mainstream school weeks | 172 | Department of Education and Skills (https://www.cso.ie) |
|  | Specialist school weeks | 339 | Special Education Section in the Department of Education and Skills (Mulkerrins, J., Personal Communication, 23rd August 2017) |
|  | Home tutor weeks | 25 | Payscale (https://www.payscale.com) |
|  | Childminder week | 30 | The National Childminding Association (https://www.childminding.ie) |
| Parental productivity losses and informal care | Productivity loss total hours | 24 | Central Statistics Office (https://www.cso.ie/) |
|  | Informal care hours per day | 24 | Central Statistics Office (https://www.cso.ie/) |

**Table A3: Italy**

|  | Cost item | Euros (2020) | Source |
| --- | --- | --- | --- |
| Speech and language therapy | PACT sessions - From the trial we know that: the average cost of a PACT session was estimated to be £264, and the total cost of the PACT intervention was £4,105 per child, on average; the mean number of sessions attended was 16 out of a maximum of 19. | 431 | see English data |
|  | Healthcare speech and language therapy sessions | 18 | Regione Emilia Romagna (https://salute.regione.emilia-romagna.it) |
| Other community health, education, and social services | General practitioner contacts | 16 | Garattiniet al (2003) (2020 values). |
|  | General practice nurse contacts | 17 | We applied the same ratio of GP nurse contacts / GP contacts in England |
|  | Health visitor contacts | 18 | Regione Emilia Romagna (https://salute.regione.emilia-romagna.it) |
|  | Community paediatrician contacts | 23 | Regione Emilia Romagna (https://salute.regione.emilia-romagna.it) |
|  | Clinical psychologist contacts | 23 | Regione Emilia Romagna (https://salute.regione.emilia-romagna.it) |
|  | Social worker contacts | 35 | Ti consiglio un lavoro website. (https://www.ticonsiglio.com) |
|  | Occupational therapist contacts | 5 | Regione Emilia Romagna (https://salute.regione.emilia-romagna.it) |
|  | Special Education Needs Coordinator contacts | 44 | Hattiva Lab Cooperativa Sociale (https://www.hattivalab.org) |
|  | Portage worker contacts | 22 | Plus Iglesias (http://www.plusdistrettoiglesias.it) |
|  | Osteopath contacts | 23 | Regione Emilia Romagna (https://salute.regione.emilia-romagna.it) |
|  | Art worker contacts | 23 | (Unit costs for GP visit Italy) * (Unit costs for this visit England)/(Unit costs for GP visit England) |
|  | Voluntary sector service contacts | 18 | (Unit costs for GP visit Italy) * (Unit costs for this visit England)/(Unit costs for GP visit England) |
|  | Voluntary sector telephone helpline calls | 18 | (Unit costs for GP visit Italy) * (Unit costs for this visit England)/(Unit costs for GP visit England) |
|  | Other community service contacts | 18 | (Unit costs for GP visit Italy) * (Unit costs for this visit England)/(Unit costs for GP visit England) |
| Hospital-based health services | Hospital nights | 1233 | Regione Emilia Romagna (https://salute.regione.emilia-romagna.it) (2020 values) |
|  | Outpatient visits | 23 | Regione Emilia Romagna (https://salute.regione.emilia-romagna.it) (2020 values) |
|  | Accident and emergency visits | 284 | Ministero della Salute (http://www.mattoni.salute.gov.it/) (2020 values) |
| Education and childcare | Mainstream nursery weeks | 82 | Comune di Modena. (https://www.comune.modena.it) |
|  | Specialist nursery weeks | 163 | ratio specialist/mainstream education 2:1 (from UK) |
|  | Mainstream playgroup weeks | 35 | https://www.laludo.it/ |
|  | Specialist playgroup weeks | 70 | ratio specialist/mainstream education 2:1 (from UK) |
|  | Mainstream school weeks | 32 | https://www.comune.modena.it |
|  | Specialist school weeks | 65 | ratio specialist/mainstream education 2:1 (from UK) |
|  | Home tutor weeks | 60 | Le tue lezioni (https://www.letuelezioni.it) |
|  | Childminder week | 74 | Childminder UK |
| Parental productivity losses and informal care | Productivity loss total hours | 15 | Luengo-Fernandez et al (2020) |
|  | Informal care hours per day | 20 | We applied the same ratio of informal care/productivity loss in England |

**Table A4: Spain**

|  | Cost item | Euros (2020) | Source |
| --- | --- | --- | --- |
| Speech and language therapy | PACT sessions - From the trial we know that: the average cost of a PACT session was estimated to be £264, and the total cost of the PACT intervention was £4,105 per child, on average; the mean number of sessions attended was 16 out of a maximum of 19. | 431 | See English data |
|  | Speech and language therapy sessions | 39 | Tarifas Para Facturación De Servicios Sanitarios Y Docentes De Osakidetza Para El Año 2020  [https://www.osakidetza.euskadi.eus](https://www.osakidetza.euskadi.eus/contenidos/informacion/osk_servic_para_empresas/es_def/adjuntos/LIBRO-DE-TARIFAS_2020_osakidetza.pdf)  (Tariff in one region in Spain: the Basque Country) |
| Other community health, education, and social services |  |  |  |
|  | General practitioner contacts | 46 | Average cost based on the tariff of the service for all (17) regions in Spain, weighted by population. (Based on PECUNIA methods) |
|  | General practice nurse contacts | 26 | Average cost based on the tariff of the service for all (17) regions in Spain, weighted by population. (Based on PECUNIA methods) |
|  | Health visitor contacts | 5 | GP costs Spain *(ratio one contact/GP contact in the UK) |
|  | Community paediatrician contacts | 40 | General practitioner contacts |
|  | Clinical psychologist contacts | 90 | Expert opinion |
|  | Social worker contacts | 36 | <https://bon.navarra.es> (Tariff in one region in Spain: Navarra; Navarra is one of the regions with highest tariffs) |
|  | Occupational therapist contacts | 27 | Expert opinion |
|  | Special Education Needs Coordinator contacts | 40 | Estimated using Wages from the national collective agreement for centres and services attending people with disabilities.  Agencia Estatal Boletín Oficial del Estado (https://www.boe.es) |
|  | Portage worker contacts | 36 | Calculated from UK estimates when applying weight based on the ratio: (Euro for one GP contact in Spain)/ (euros for one GP contact in the UK) |
|  | Osteopath contacts | 46 | Calculated from UK estimates when applying weight based on the ratio: (Euro for one GP contact in Spain)/ (euros for one GP contact in the UK) |
|  | Art worker contacts | 57 | Calculated from UK estimates when applying weight based on the ratio: (Euro for one GP contact in Spain)/ (euros for one GP contact in the UK) |
|  | Voluntary sector service contacts | 46 | Calculated from UK estimates when applying weight based on the ratio: (Euro for one GP contact in Spain)/ (euros for one GP contact in the UK) |
|  | Voluntary sector telephone helpline calls | 46 | Calculated from UK estimates when applying weight based on the ratio: (Euro for one GP contact in Spain)/ (euros for one GP contact in the UK) |
|  | Other community service contacts | 46 | Calculated from UK estimates when applying weight based on the ratio: (Euro for one GP contact in Spain)/ (euros for one GP contact in the UK) |
| Hospital-based health services | Hospital nights | 735 | Ministerio de Sanidad SSeI. Diagnósticos Principales. (http://pestadistico.inteligenciadegestion.msssi.es) |
|  | Outpatient visits | 134 | Generalitat de Catalunya. (http://portaldogc.gencat.cat) |
|  | Accident and emergency visits | 190 | Generalitat de Catalunya. (http://portaldogc.gencat.cat) |
| Education and childcare | Mainstream nursery weeks | 300 | Expert opinion |
|  | Specialist nursery weeks | -- | Not available in Spain |
|  | Mainstream playgroup weeks | -- | Not available in Spain |
|  | Specialist playgroup weeks | -- | Not available in Spain |
|  | Mainstream school weeks | 400 | Expert opinion |
|  | Specialist school weeks | 500 | Expert opinion |
|  | Home tutor weeks | -- | Not available in Spain |
|  | Childminder week | -- | Not available in Spain |
| Parental productivity losses and informal care | Productivity loss total hours | 10 | Luengo-Fernandez wt al (2020) |
|  | Informal care hours per day | 14 | Productivity loss Spain *(ratio informal care/productivity loss UK) |

**References for appendix 2**

Agencia Estatal Boletín Oficial del Estado website. Available at: <https://www.boe.es>

Bishopthorpe Preschool Playgroup website. Available at: <https://www.bishopthorpe-playgroup.org.uk>

Byford S, Cary M, Barrett B, Aldred CR, Charman T, Howlin P, Hudry K, Leadbitter K, Le Couteur A, McConachie H, Pickles A, Slonims V, Temple KJ, Green J; PACT Consortium. Cost-effectiveness analysis of a communication-focused therapy for pre-school children with autism: results from a randomised controlled trial. BMC Psychiatry. 2015 Dec 21;15:316.

Central Statistics Office website. Available at: <https://www.cso.ie/>

Comune di Modena website. Available at: <https://www.comune.modena.it>

Curtis LA, Burns A. Unit Costs of Health & Social Care 2020. Unit Costs of Health and Social Care. PSSRU, University of Kent.

Department for Education. Available at: https://www.gov.uk/government/organisations/department-for-education

Garattini L, Castelnuovo E, Lanzeni D, Viscarra C. Durata e costo delle visite in medicina generale: il progetto DYSCO. Farmeconomia e percorsi terapeutici. 2002; 4 (2): 109-114.

Generalitat de Catalunya. Available at: http://portaldogc.gencat.cat

Government of Ireland website. Available at: https://www.gov.ie

Hattiva Lab Cooperativa Sociale website. Available at: https://www.hattivalab.org

Health Careers. Available at: <https://www.healthcareers.nhs.uk>

Indeed Ireland website. Available at: https://ie.indeed.com/

Irish Nurses & Midwives Organisation website. Available at: https://www.inmo.ie

Le tue lezioni website. Available at: <https://www.letuelezioni.it>

Luengo-Fernandez R, Violato M, Candio P, Leal J. Economic burden of stroke across Europe: A population-based cost analysis. Eur Stroke J 2020;5(1):17-25.

Money Helper website. Available at: [https://www.moneyhelper.org.uk/en?source=mas#](https://www.moneyhelper.org.uk/en?source=mas)

Ministerio de Sanidad SSeI. Diagnósticos Principales website. Available at:

http://pestadistico.inteligenciadegestion.msssi.es

Ministero della Salute website. Available at: <http://www.mattoni.salute.gov.it/>

National Career Service website. Available at: <https://nationalcareers.service.gov.uk>

National Health System website. Available at: <https://www.nhs.uk>

Navarra region website. Available at: <https://bon.navarra.es>

NHS England. National Cost Collection for the NHS. Available at: <https://www.england.nhs.uk/national-cost-collection/>.

Office of National Statistics. Available at: <https://www.ons.gov.uk/>

Office of the Regional Director of Operations. Available at: https://www.hse.ie

Payscale website. Available at: https://www.payscale.com

Prospect UK website. Available at: <https://www.prospects.ac.uk/>

Plus Iglesias website. Available at: https://www.plusdistrettoiglesias.it/it/index.html

Regione Emilia Romagna webiste. Available at: <https://salute.regione.emilia-romagna.it>

Tarifas Para Facturación De Servicios Sanitarios Y Docentes De Osakidetza Para El Año 2020.

Available at: [https://www.osakidetza.euskadi.eus](https://www.osakidetza.euskadi.eus/contenidos/informacion/osk_servic_para_empresas/es_def/adjuntos/LIBRO-DE-TARIFAS_2020_osakidetza.pdf)

The Institute for Fiscal Studies (IFS) website. Available at: <https://ifs.org.uk/>

The National Childminding Association website. Available at: https://www.childminding.ie/

The Osteopathic Council of Ireland website. Available at: https://www.osteopathy.ie/

ThirdSector website. Available at: <https://www.thirdsector.co.uk/>

Ti consiglio un lavoro website. Available at: https://www.ticonsiglio.com

**Appendix 3: Sensitivity analysis 1**

**Figure A1: Difference in total societal costs between groups for England (mean of intervention minus control at 13 months and 6 years; Euros)**

For each category of costs, the relative unit cost estimates were varied by a given amount (+/-20%; +/-30% and +/-50%).

**Figure A2: Difference in total societal costs between groups for Ireland (mean of intervention minus control at 13 months and 6 years; Euros)**

For each category of costs, the relative unit cost estimates were varied by a given amount (+/-20%; +/-30% and +/-50%).

**Figure A3: Difference in total societal costs between groups for Italy (mean of intervention minus control at 13 months and 6 years; Euros)**

For each category of costs, the relative unit cost estimates were varied by a given amount (+/-20%; +/-30% and +/-50%).

**Figure A4: Difference in total societal costs between groups for Spain (mean of intervention minus control at 13 months and 6 years; Euros)**

For each category of costs, the relative unit cost estimates were varied by a given amount (+/-20%; +/-30% and +/-50%).

**Appendix 4: Sensitivity analysis 2**

**Table A1: Total costs societal (Euros) per person at 6 years (England)**

|  | PACT + TAU | TAU | PACT + TAU minus TAU |
| --- | --- | --- | --- |
|  | **Mean costs (euros)** | **Mean costs (euros)** | **The difference in costs (euros)** |
| PACT | 6,198 | 0 | 6,198 |
| Healthcare speech and language therapy | 3,285 | 3,145 | 139 |
| Other community health, education, and social services | 8,270 | 5,945 | 2,324 |
| Hospital-based health services | 3,890 | 5,623 | - 1,732 |
| Education and childcare | 39,460 | 45,845 | - 6,384 |
| Parental productivity losses | 3,531 | 1,995 | 1,535 |
| Parental informal care | 348,877 | 386,468 | - 37,590 |
| Total all above | **413,514** | **449,023** | **- 35,509** |

Note: Cost estimates were calculated by multiplying the use of resources (Byford, et al. 2015) by unit cost estimates (Supplementary material appendix 1). Temporal extrapolation techniques (Bojke et al., 2017) were used to build economic estimates for 6 years.

**Table A2: Total societal costs (Euros) at 6 years (Ireland)**

|  | PACT + TAU | TAU | PACT + TAU minus TAU |
| --- | --- | --- | --- |
|  | **Mean costs (euros)** | **Mean costs (euros)** | **The difference in costs (euros)** |
| PACT | 6,198 | 0 | 6,198 |
| Healthcare speech and language therapy | 3,271 | 3,132 | 139 |
| Other community health, education, and social services | 7,728 | 5,357 | 2,371 |
| Hospital-based health services | 2,231 | 5,042 | - 2,810 |
| Education and childcare | 61,408 | 62,905 | - 1,496 |
| Parental productivity losses | 4,873 | 2,754 | 2,119 |
| Parental informal care | 52,182 | 390,129 | - 37,946 |
| Total all above | 437,894 | 469,320 | - 31,426 |

Note: Cost estimates were calculated by multiplying the use of resources (Byford, et al. 2015) by unit cost estimates (Supplementary material appendix 1). Temporal extrapolation techniques (Bojke et al., 2017) were used to build economic estimates for 6 years.

**Table A3: Total societal costs (Euros) at 6 years (Italy)**

|  | PACT + TAU | TAU | PACT + TAU minus TAU |
| --- | --- | --- | --- |
|  | **Mean costs (euros)** | **Mean costs (euros)** | **The difference in costs (euros)** |
| PACT | 6,198 | 0 | 6,198 |
| Healthcare speech and language therapy | 1,219 | 1,167 | 51 |
| Other community health, education and social services | 2,616 | 1,808 | 807 |
| Hospital-based health services | 1,509 | 5,138 | - 3,628 |
| Education and childcare | 19,662 | 21,052 | - 1,390 |
| Parental productivity losses | 3,058 | 1,728 | 1,330 |
| Parental informal care | 288,911 | 320,040 | - 31,129 |
| Total all above | 323,174 | 350,935 | - 27,761 |

Note: Cost estimates were calculated by multiplying the use of resources (Byford, et al. 2015) by unit cost estimates (Supplementary material appendix 1). Temporal extrapolation techniques (Bojke et al., 2017) were used to build economic estimates for 6 years.

**Table A4: Total societal costs (Euros) at 6 years (Spain)**

|  | PACT + TAU | TAU | PACT + TAU minus TAU |
| --- | --- | --- | --- |
|  | **Mean costs (euros)** | **Mean costs (euros)** | **The difference in costs (euros)** |
| PACT | 6,194 | - | 6,194 |
| Healthcare speech and language therapy | 2,641 | 2,529 | 112 |
| Other community health, education, and social services | 6,051 | 4,143 | 1,908 |
| Hospital-based health services | 2,405 | 4,415 | - 2,010 |
| Education and childcare | 112,041 | 114,408 | - 2,366 |
| Parental productivity losses | 2,099 | 1,186 | 912 |
| Parental informal care | 206,282 | 228,508 | - 22,226 |
| Total all above | 337,716 | 355,191 | - 17,474 |

Note: Cost estimates were calculated by multiplying the use of resources (Byford, et al. 2015) by unit cost estimates (Supplementary material appendix 1). Temporal extrapolation techniques (Bojke et al., 2017) were used to build economic estimates for 6 years.

**References for appendix 4**

Byford S, Cary M, Barrett B, Aldred CR, Charman T, Howlin P, Hudry K, Leadbitter K, Le Couteur A, McConachie H, Pickles A, Slonims V, Temple KJ, Green J; PACT Consortium. Cost-effectiveness analysis of a communication-focused therapy for pre-school children with autism: results from a randomised controlled trial. BMC Psychiatry. 2015 Dec 21;15:316.

Bojke L, Manca A, Asaria M, Mahon R, Ren S, Palmer S. How to Appropriately Extrapolate Costs and Utilities in Cost-Effectiveness Analysis. Pharmacoeconomics. 2017 Aug;35(8):767-776.
